# PRMT2 silencing regulates macrophage polarization through activation of STAT1 or inhibition of STAT6

**DOI:** 10.1186/s12865-023-00593-w

**Published:** 2024-01-03

**Authors:** Ting Liu, Yinjiao Li, Muqiu Xu, Hongjun Huang, Yan Luo

**Affiliations:** Department of Anesthesiology, Ruijin Hospital, Shanghai Jiao Tong University School of Medicine, Shanghai, 200025 China

**Keywords:** PRMT2, Macrophage polarization, M1/M2, STAT1/STAT6

## Abstract

**Background:**

Macrophages play significant roles in innate immune responses and are heterogeneous cells that can be polarized into M1 or M2 phenotypes. PRMT2 is one of the type I protein arginine methyltransferases involved in inflammation. However, the role of PRMT2 in M1/M2 macrophage polarization remains unclear. Our study revealed the effect and mechanism of PRMT2 in macrophage polarization.

**Methods:**

Bone marrow-derived macrophages (BMDMs) were polarized to M1 or M2 state by LPS plus murine recombinant interferon-γ (IFN-γ) or interleukin-4 (IL-4). Quantitative polymerase chain reaction (qPCR), western blot and flow cytometry (FCM) assay were performed and analyzed markers and signaling pathways of macrophage polarization.

**Results:**

We found that PRMT2 was obviously upregulated in LPS/IFN-γ-induced M1 macrophages, but it was little changed in IL-4-induced M2 macrophages. Furthermore, PRMT2 konckdown increased the expression of M1 macrophages markers through activation of STAT1 and decreased the expression of M2 macrophages markers through inhibition of STAT6.

**Conclusions:**

PRMT2 silencing modulates macrophage polarization by activating STAT1 to promote M1 and inhibiting STAT6 to attenuate the M2 state.

**Supplementary Information:**

The online version contains supplementary material available at 10.1186/s12865-023-00593-w.

## Background

Macrophages are essential in tissue homeostasis and inflammation, which perform vital tissue-specific functions and protect the organism from infection [1]. Additionally, macrophage populations vary among tissues, with osteoclasts and microglia as noted examples [2]. Notably, the diversity and plasticity of macrophage lineages are crucial aspects of their function. Murine bone marrow-derived macrophages (BMDMs) are usually used to research macrophage functions in vitro, which can be generated from bone marrow to mature macrophages by the addition of recombinant murine macrophage colony-stimulating factor (M-CSF) [3]. Macrophages can also be induced into different phenotypes when undergoing classical or alternative activation, called M1 (induced by LPS/IFN-γ) or M2 macrophages (induced by IL-4/IL-13) in vitro, respectively. Evidence has shown that M1 phenotype is tightly associated with pro-inflammatory responses, yet M2 state plays a critical role in anti-inflammatory responses [4, 5]. M1-type pro-inflammatory markers include tumor necrosis factor α (TNF-α), IL-1β, IL-6, inducible nitric oxide synthase (iNOS), and CD86, whereas IL-10, IL-13, found in inflammatory zone 1 (FIZZ1), Arginase 1 (Arg1), transforming growth factor β (TGF-β) and CD206 are M2-type anti-inflammatory markers [6, 7].

Post-translational modifications (PTMs) refer to chemical modifications during the post-translational formation of proteins. Specifically, during the process of protein synthesis, certain biochemically functional groups (phosphate groups, acetyl groups, lipids, carbohydrates, etc.) can be covalently bound to the protein backbone or side chains by the action of modifying enzymes that change their biophysical properties, thus affecting their localization, stability, interactions and lying at the heart of the fields of epigenetics and signal transduction [8]. Protein arginine methylation is a type of PTM catalyzed by protein arginine methyltransferases (PRMTs). Additionally, they can modify target proteins through methylation of the guanidinium nitrogen atom to the arginine residue [9]. Notably, PRMTs modulate basic cellular processes, including transcription, RNA processing, signal transduction cascades, and DNA damage responses [10–12]. PRMTs include PRMT1 to 9 and can produce MMA (mono-methyl-arginine), aDMA (asymmetric dimethyl-arginine), or sDMA (symmetric dimethyl-arginine) [13]. Based on the final methylarginine product generated, PRMTs are classified into three categories: type II PRMTs, including PRMT5 and PRMT9 catalyzing the generation of MMA and sDMA; type III PRMT, including PRMT7 catalyzing the generation of MMA, and the remaining are type I PRMTs catalyzing the generation of MMA and aDMA [14, 15].

PRMT2 is unique in the PRMT family for containing an Src Homology 3 (SH3) domain. It has been illustrated that it acts as a transcriptional coactivator for nuclear hormone receptors and is consequently associated with breast cancer [16]. A previous study indicated that PRMT2 was associated with the development of glioblastoma [17]. Higher PRMT2 expression is observed in intestinal specimens from patients with Crohn’s disease and ulcerative colitis, additionally, PRMT2 represses the SOCS3 promoter via histone H3R8 asymmetric dimethylation, thereby promoting the development of DSS-induced colitis [18]. PRMT2 has also been described to increase the accumulation of IκB-α (NF-κB blocker) in the nucleus, which inhibits NF-κB-dependent transcription [19]. Furthermore, in lung tissue and macrophages, reduced PRMT2 expression could increase their responsiveness to LPS and promotes the expression of inflammatory cytokines [20]. It has also been shown that PRMT2 is involved in the regression of diabetic atherosclerosis, and PRMT2 deficiency fosters the expression of genes associated with cytokine signaling and inflammatory pathways in atherosclerotic plaque CD68^+^ cells [21]. In addition, LPS induces arginine methylation of TLR4 and IRF3 in RAW264.7 via PRMT2, thereby promoting IFN-β production [22]. Thus, the expression of PRMT2 in macrophages is closely related to inflammation. However, few studies have investigated the relationship between PRMT2 and macrophage polarization and its mechanisms.

In this study, we screened and found that PRMT2 could regulate macrophage polarization. Briefly, PRMT2 silencing promotes M1 polarization by STAT1 activation and attenuates M2 polarization through STAT6 inhibition.

## Results

### PRMT2 is upregulated in LPS- or LPS/IFN-γ-treated BMDMs

**Fig. 1:**
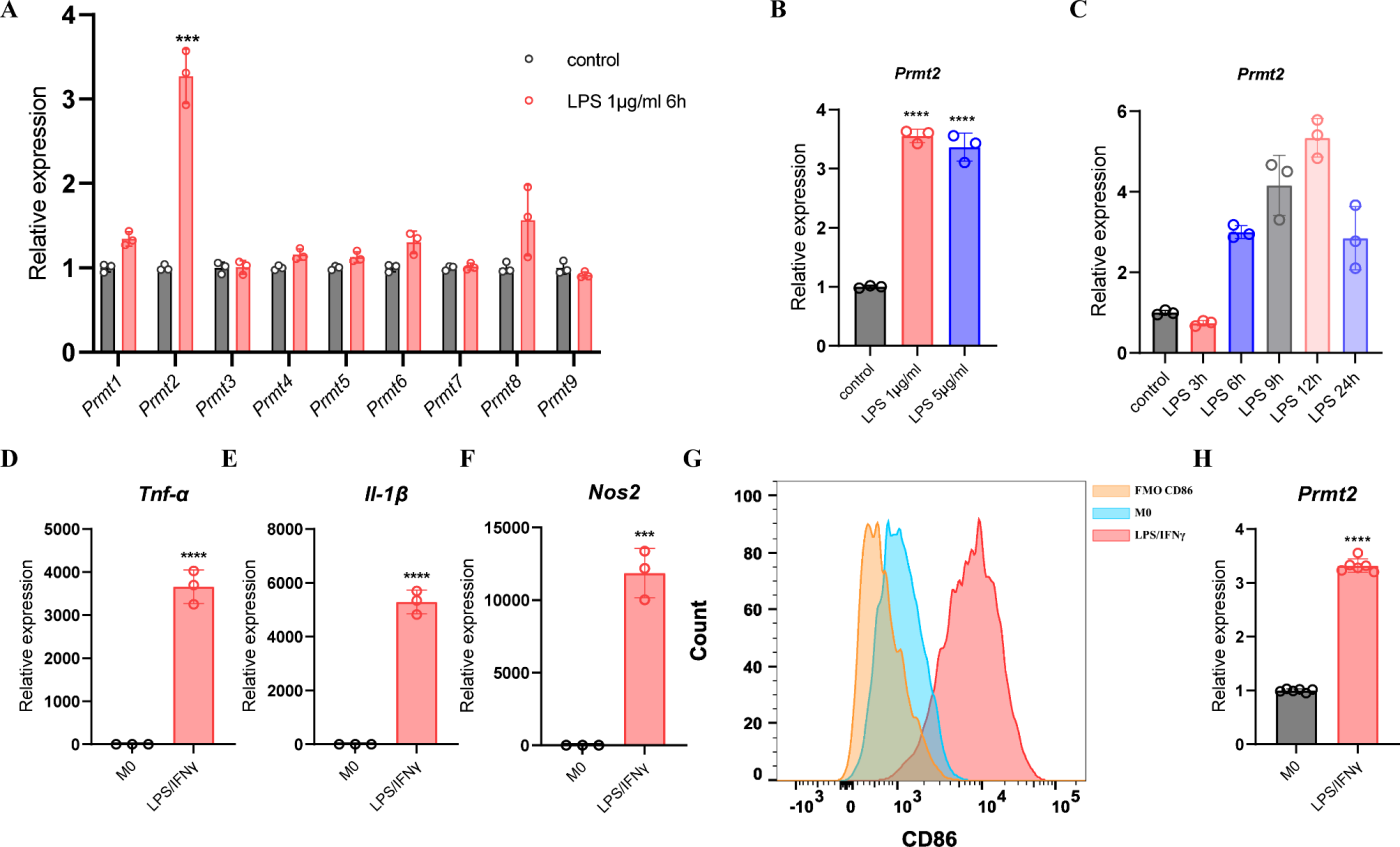
PRMT2 is upregulated in LPS- or LPS/IFN-γ-treated BMDMs. **(A)** Quantitative polymerase chain reaction (qPCR) analysis of *Prmts* expression in BMDMs treated with LPS (1 µg/ml) for 6 h. **(B)** qPCR analysis of *Prmt2* expression in BMDMs treated with LPS (1 µg/ml and 5 µg/ml) for 6 h. **(C)** qPCR analysis of *Prmt2* expression in BMDMs treated with LPS (1 µg/ml) for the indicated times. **(D-G)** qPCR analysis of *Tnf-α* **(D)**, *Il-1β* **(E)**, and *Nos2* **(F)** expression and Flow cytometry (FCM) analysis of CD86 **(G)** in BMDMs treated with LPS (100 ng/ml) plus IFN-γ (20 ng/ml) for 24 h. FMO, fluorescence minus one. **(H)** qPCR analysis of *Prmt2* expression in BMDMs treated with LPS (100 ng/ml) plus IFN-γ (20 ng/ml) for 24 h. 18s rRNA was used as an endogenous reference for qPCR. Data are representative of three independent experiments (mean ± SD). ***p < 0.001, ****p < 0.0001

To investigate the potential roles of PRMTs in macrophage polarization, we treated mature BMDMs with LPS or LPS plus IFN-γ to induce the M1 phenotype. As depicted in Fig. 1A, the expression of *Prmt2* mRNA was considerably increased by LPS in BMDMs. Consistent with this result, LPS treatment enhanced *Prmt2* expression in BMDMs with different doses and times (Fig. 1B, C). We next detected the mRNA level of M1 markers and found that *Tnf-α*, *IL-1β*, and *Nos2* expression were elevated obviously after LPS/IFN-γ stimulation (Fig. 1D-F). Similarly, surface marker CD86 was also significantly increased in LPS/IFN-γ-stimulated BMDMs by flow cytometry (Fig. 1G). Furthermore, we confirmed that the expression of *Prmt2* was enhanced in LPS/IFN-γ-treated BMDMs (Fig. 1H). Therefore, these data suggest that PRMT2 is upregulated by LPS or LPS/IFN-γ in BMDMs, which may act as a regulator of macrophage polarization.

### PRMT2 silencing promotes M1 polarization

**Fig. 2:**
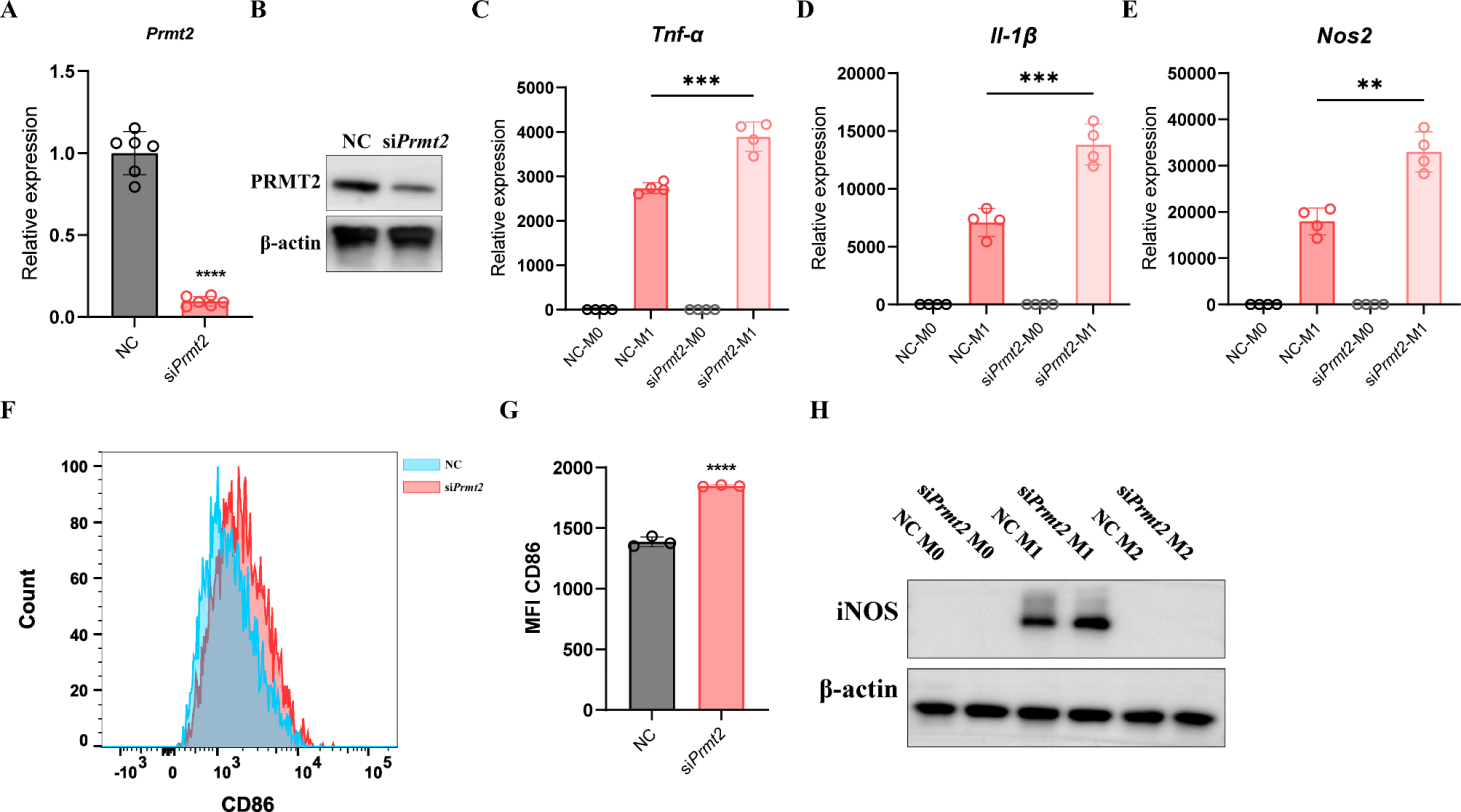
PRMT2 silencing promotes M1 polarization. **(A, B)** qPCR analysis of *Prmt2* mRNA **(A)** expression and immunoblot analysis of PRMT2 protein **(B)** in BMDMs transfected with *Prmt2* siRNA for 48 h. **(C-H)** qPCR analysis of *Tnf-α* **(C)**, *Il-1β* **(D)**, and *Nos2* **(E)** mRNA expression and FCM analysis of CD86 **(F)** expression in BMDMs transfected with *Prmt2* siRNA for 48 h and then treated with LPS (100 ng/ml) plus IFN-γ (20 ng/ml) for 24 h. **(G)** Quantification of mean fluorescence intensity of CD86 in **(F)**. **(H)** Immunoblot analysis of iNOS in BMDMs transfected with *Prmt2* siRNA for 48 h and then treated with LPS (100 ng/ml) plus IFN-γ (20 ng/ml) or IL-4 (20 ng/ml) for 24 h. 18s rRNA was used as an endogenous reference for qPCR. Data are representative of three independent experiments (mean ± SD). **p < 0.01, ***p < 0.001, ****p < 0.0001

Since PRMT2 is elevated in M1 macrophages, we next silenced endogenous PRMT2 expression in BMDMs using *Prmt2*-specific siRNA and confirmed that *Prmt2* mRNA and PRMT2 protein expression were notably reduced in PRMT2-knockdown cells (Fig. 2A, B). To explore the effect of PRMT2 during macrophage polarization, we exposed PRMT2-silenced BMDMs to LPS/IFN-γ induces M1 phenotype. Then, M1 macrophage markers, including TNF-α, IL-1β, iNOS, and CD86, were examined. As presented in Fig. 2C-E, *Tnf-α*, *Il-1β*, and *Nos2* mRNA levels were significantly increased in PRMT2-knockdown M1 macrophages compared to controls. Similarly, CD86 expression was also increased in PRMT2-knockdown M1 macrophages by flow cytometry (Fig. 2F, G). LPS/IFN-γ treatment also enhanced iNOS protein levels in PRMT2-silenced macrophages (Fig. 2H). Thus, these results indicate that PRMT2 silencing promotes M1 polarization.

### PRMT2 silencing attenuates M2 polarization

**Fig. 3:**
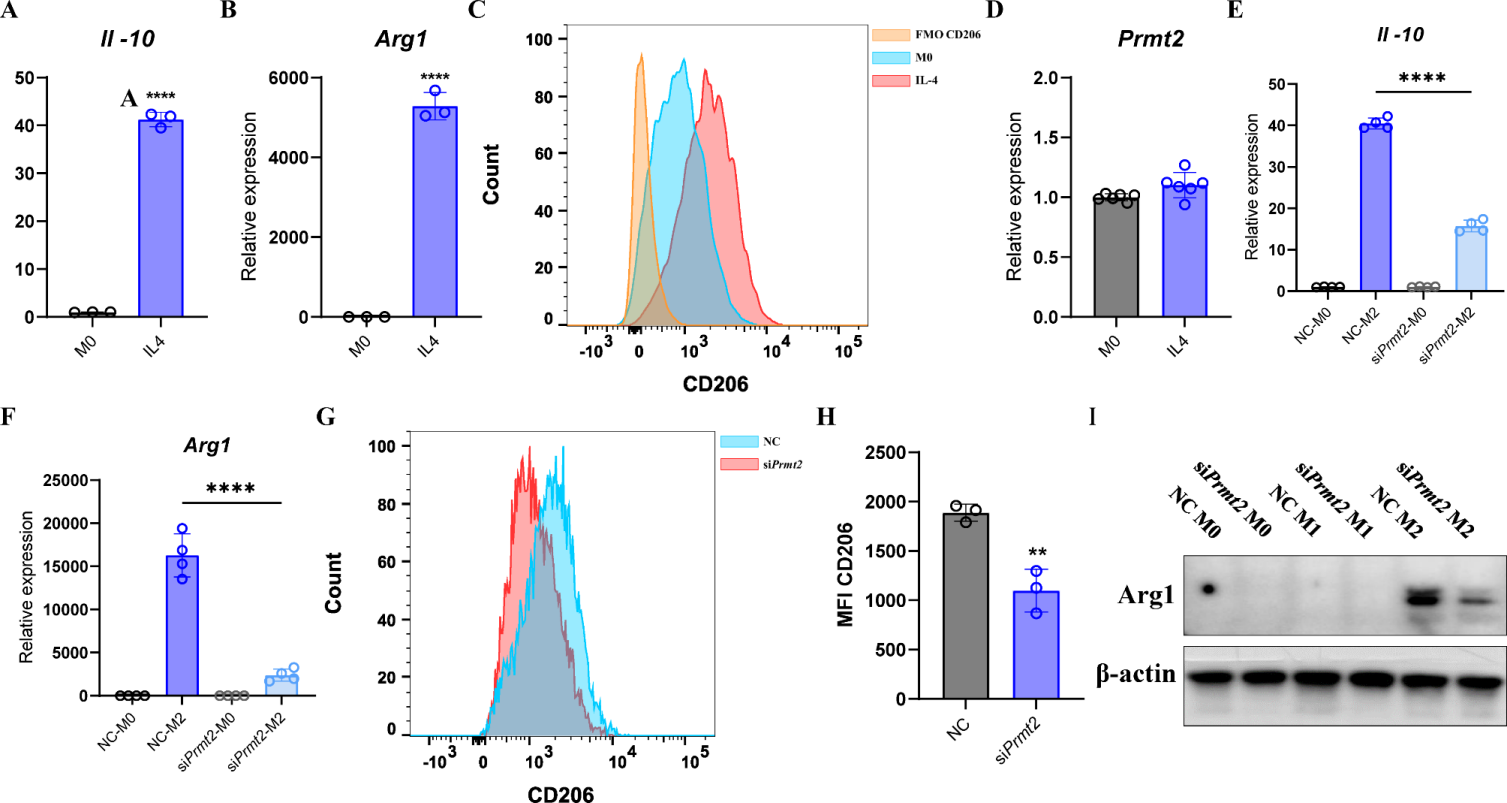
PRMT2 silencing attenuates M2 polarization. **(A-C)** qPCR analysis of *Il-10* **(A)** and *Arg1* **(B)** expression and FCM analysis of CD206 **(C)** in BMDMs treated with IL-4 (20 ng/ml) for 24 h. **(D)** qPCR analysis of *Prmt2* expression in BMDMs treated with IL-4 (20 ng/ml) for 24 h. **(E-H)** qPCR analysis of *Il-10* **(E)** and *Arg1***(F)** expression and FCM analysis of CD206 (**G**) in BMDMs transfected with *Prmt2* siRNA for 48 h and then treated with IL-4 (20 ng/ml) for 24 h. **(H)** Quantification of mean fluorescence intensity of CD206 in (**G**). **(I)** Immunoblot analysis of ARG1 in BMDMs transfected with *Prmt2* siRNA for 48 h and then treated with LPS (100 ng/ml) plus IFN-γ (20 ng/ml) or IL-4 (20 ng/ml) for 24 h. 18s rRNA was used as an endogenous reference for qPCR. Data are representative of three independent experiments (mean ± SD). **p < 0.01, ****p < 0.0001

It is clear that PRMT2 knockdown promotes M1 polarization. To determine whether PRMT2 silencing affects M2 polarization, we treated BMDMs with IL-4 to induce M2 polarization and demonstrated that the transcription of *Il-10* and *Arg1* was successfully induced in IL-4-treated macrophages (Fig. 3A, B). Consistent with these results, FCM showed that IL-4 treatment notably increased the expression of surface marker CD206 in BMDMs (Fig. 3C). However, unlike M1 macrophages, compared to M0 macrophages, the expression of *Prmt2* was not changed in M2 macrophages (Fig. 3D). Regardless, the expression of *Il-10* and *Arg1* were reduced in PRMT2-knockdown M2 macrophages (Fig. 3E, F). Simultaneously, CD206 expression was reduced by the detection of flow cytometry (Fig. 3G, H). As predicted, IL-4 treatment decreased ARG1 protein level in PRMT2-knockdown macrophages (Fig. 3I). Therefore, silencing of PRMT2 attenuates M2 polarization.

### PRMT2 silencing promotes M1 polarization through STAT1 activation

**Fig. 4:**
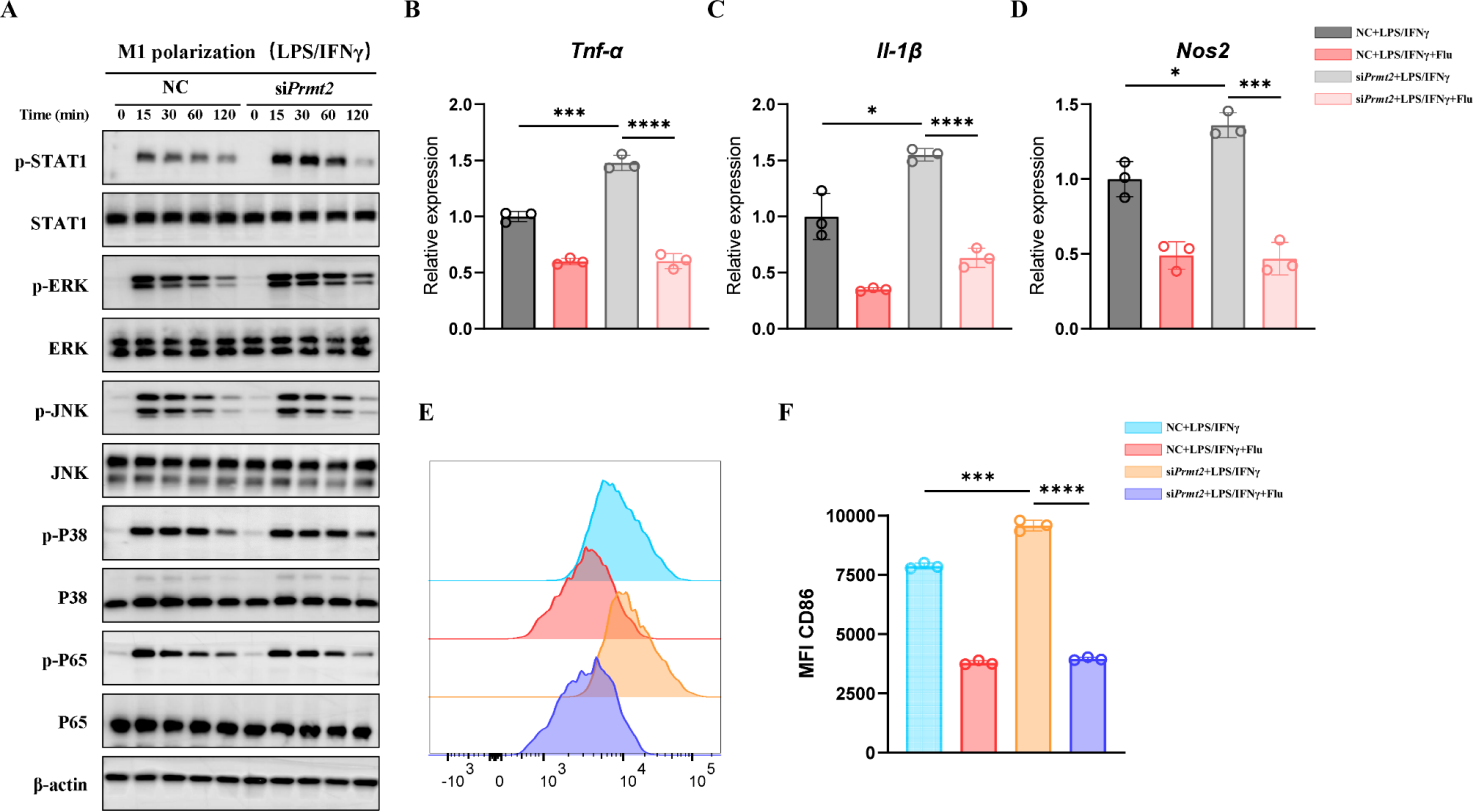
PRMT2 silencing promotes M1 polarization through STAT1 activation. **(A)** Immunoblot analysis of phosphorylated STAT1, ERK, JNK, P38, and P65 or total proteins in lysates of BMDMs transfected with *Prmt2* siRNA for 48 h and then treated with LPS (100 ng/ml) plus IFN-γ (20 ng/ml) for the indicated times. **(B-E)** qPCR analysis of *Tnf-α* **(B)**, *Il-1β* **(C)**, and *Nos2* **(D)** expression and FCM analysis of CD86 **(E)** in BMDMs transfected with *Prmt2* siRNA for 48 h and then pretreated with STAT1 inhibitor Fludarabine (10 µM) for 1 h following by LPS (100 ng/ml) plus IFN-γ (20 ng/ml) stimulation for 24 h. **(F)** Quantification of mean fluorescence intensity of CD86 in **(E).** 18s rRNA was used as an endogenous reference for qPCR. Data are representative of three independent experiments (mean ± SD). *p < 0.05, ***p < 0.001, ****p < 0.0001

Next, we explored how PRMT2 modulated macrophage polarization. It is well known that signal transducer and activator of transcription 1 (STAT1), MAPK, and NF-κB pathways serve critical roles in inflammation and macrophage polarization [23]. To reveal whether PRMT2 knockdown affects these pathways in macrophages polarized toward the M1 state, we treated BMDMs with LPS/IFN-γ and found that phosphorylated ERK (p-ERK), p-JNK, p-P38, and p-P65 were only slightly influenced by PRMT2 silencing (Fig. 4A). However, STAT1 phosphorylation increased dramatically in PRMT2-knockdown BMDMs following LPS/IFN-γ treatment (Fig. 4A). To further validate the activation of STAT1 affecting M1 macrophage polarization in PRMT2-silenced BMDMs, the STAT1 inhibitor Fludarabine was administrated. Notably, Fludarabine blocked LPS/IFN-γ-induced elevation of *Tnf-α*, *Il-1β*, and *Nos2* expression in PRMT2-silenced cells (Fig. 4B-D). Likewise, flow cytometry showed that CD86 increase was also blocked by Fludarabine in LPS/IFN-γ-treated PRMT2-knockdown macrophages (Fig. 4E, F). Collectively, PRMT2 silencing promotes M1 polarization through the activation of STAT1 signaling.

### PRMT2 silencing attenuates M2 polarization through STAT6 inhibition

**Fig. 5:**
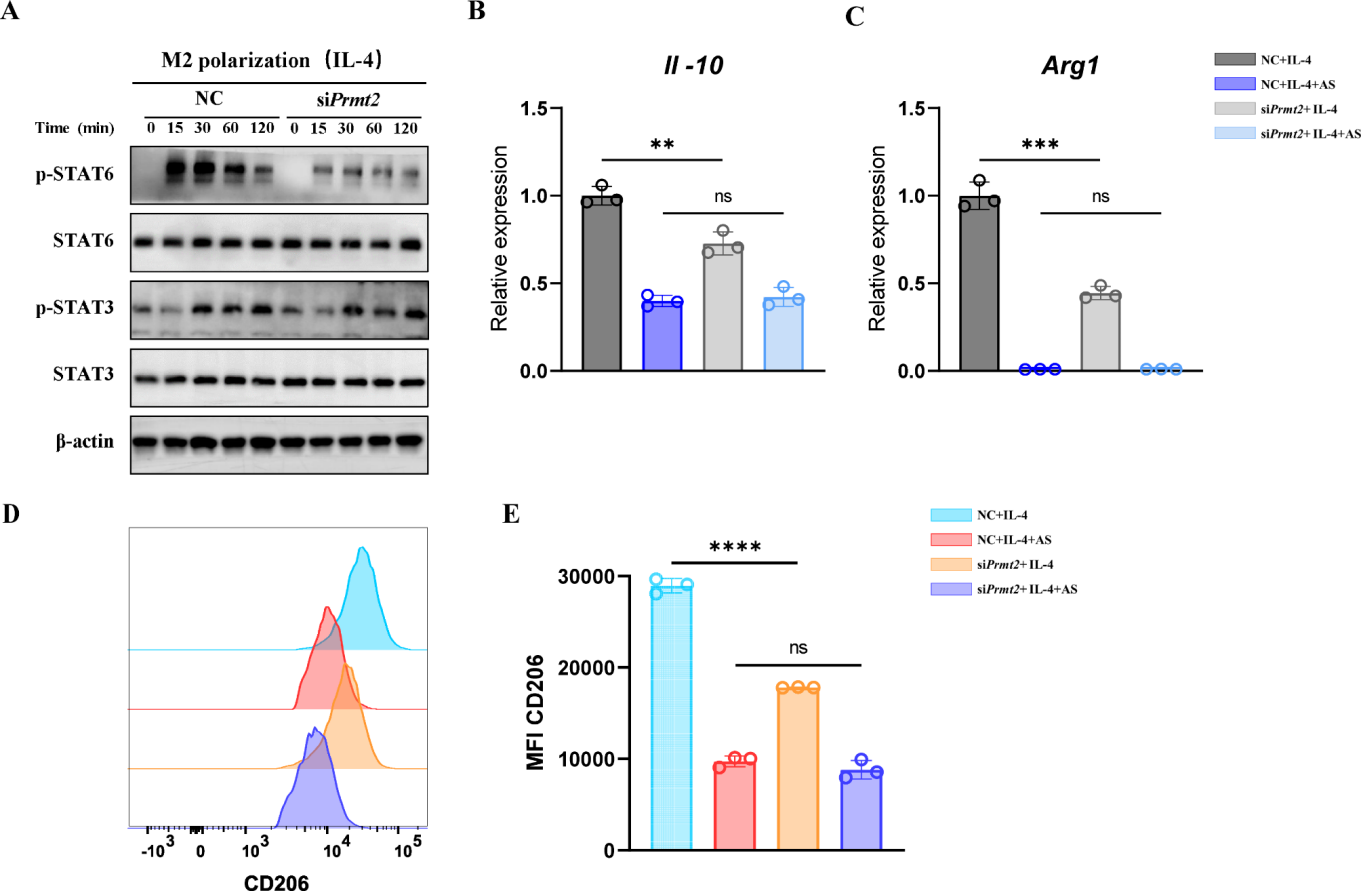
PRMT2 silencing attenuates M2 polarization through STAT6 inhibition. **(A)** Immunoblot analysis of phosphorylated STAT3 and STAT6 or total proteins in lysates of BMDMs transfected with *Prmt2* siRNA for 48 h and then treated with IL-4 (20 ng/ml) for the indicated times. **(B-D)** qPCR analysis of *Il-10* **(B)** and *Arg1* **(C)** expression and FCM analysis of CD206 (**D**) in BMDMs transfected with *Prmt2* siRNA for 48 h and then pretreated with STAT6 inhibitor AS1517499 (1 µM) for 1 h following by IL-4 (20 ng/ml) stimulation for 24 h. **(E)** Quantification of mean fluorescence intensity of CD206 in (**D**). 18s rRNA was used as an endogenous reference for qPCR. Data are representative of three independent experiments (mean ± SD). **p < 0.01, ***p < 0.001, ****p < 0.0001. ns, not significant

To further determine the mechanism of PRMT2-mediated M2 polarization, we detected phosphorylated STAT3 (p-STAT3) and p-STAT6, which are reported to regulate M2 polarization [24]. As shown in Fig. 5A, IL-4-induced STAT6 phosphorylation was noticeably reduced in PRMT2-knockdown BMDMs. Most importantly, the reduction of *Il-10* and *Arg1* expression caused by PRMT2 silencing could be counteracted by the STAT6 inhibitor, AS1517499. Specifically, these M2 markers were largely unaffected between controls and PRMT2 silencing with the addition of AS1517499 (Fig. 5B, C). Expectedly, the same conclusion was obtained from the flow cytometry detection of CD206 (Fig. 5D, E). Taken together, PRMT2 silencing attenuates M2 polarization by inhibition of STAT6 signaling.

## Discussion

In the present study, we provide evidence to demonstrate that PRMT2 modulates macrophage polarization in vitro. PRMT2 belongs to PRMTs and can act as a coactivator of several nuclear hormone receptors [25]. A previous study found that PRMT2 promotes apoptosis by suppressing NF-κB-dependent transcription [19]. Moreover, PRMT2 plays a role in LPS-induced pulmonary inflammation and airway distress syndrome by regulating NF-κB [20]. In a study, utilizing lung tissue and macrophages, the expression of PRMT2 was downregulated with LPS, allowing NF-κB to bind to the promoter of its target gene. The absence of PRMT2 facilitates NF-κB accumulation in the nucleus after LPS treatment and then increases the production of TNF-α and IL-6, resulting in an inflammatory response. However, in our study, we demonstrate that PRMT2 is upregulated in LPS- or LPS/IFN-γ-treated BMDMs, and PRMT2 silencing has little influence on NF-κB activation. Our findings indicate that PRMT2 regulation of macrophage polarization might be an intrinsic mechanism of PRMT2-mediated inflammatory regulation. Our study is also consistent with Beyza Vurusaner and his/her colleagues’ research that loss of PRMT2 in BMDMs shows an increased expression of pro-inflammatory genes toward LPS and compatibility with a decrease in genes of inflammation resolving in response to IL-4 [21].

Signal transducer and activator of transcription (STAT) proteins consist of STAT1, 2, 3, 4, 5a, 5b, and 6, which are transcription factors localized in the cytoplasm [26]. STATs are widely distributed in the cytoplasm at rest and translocate into the nucleus in response to stimulation of extracellular ligands, cytokines, and growth factors, which participate in biological processes such as cell proliferation, differentiation, migration, and apoptosis [27]. Evidence suggests that IFN-γ activates JAK-STAT1 signaling and promotes STAT1 phosphorylation to promote the polarization of M1-type macrophage [28]. Meanwhile, tyrosine phosphorylation and activation of STAT6 mediates M2 polarization via transcriptional activation of M2 phenotype-specific genes [29]. In STAT6-overexpressing macrophages, M2 genes enhanced expression [30], whereas the ablation of STAT6 eliminated M2 gene expression [31]. Additionally, macrophage plasticity can be driven by different stimuli. With IFN-γ stimulation, STAT1 binds many regulatory elements linked to IL-4-inducible genes. Similarly, STAT6 binds a significant portion of the regulatory elements of IFN-γ activated genes in response to IL-4 [32]. Many STAT1- and STAT6-binding events generally occur on overlapping or adjacent genomic regions. The data imply a reciprocal inhibitory role of STAT1 and STAT6 in the genomic organization. Notably, PRMT2 silencing may affect these regions and thus modify STAT1 activation and STAT6 inhibition during macrophage polarization. In addition, the mutual inhibition between STAT1 and STAT6 may be due to the ability of STAT1 to transcribe some regulatory factors to regulate STAT6, and vice versa.

JAK-STAT signaling is regulated by arginine methylation. It includes the mechanism of PRMT1 binding and methylating STAT1 on Arg31 [33]. Arg31 is conserved in both STAT3 and STAT6, which can also undergo arginine methylation [34, 35]. Importantly, PRMT1 catalyzes STAT3 arginine methylation to facilitate the transcriptional activity of STAT3-targeted astrocyte-specific genes and then promotes astrocyte differentiation of neural stem/precursor cells [36]. In germinal center B cells, PRMT5 is a JAK-binding and JAK1- and JAK3-related arginine methyltransferase that regulates STAT6 activity in response to IL-4 stimulation [37]. In another study, authors identified that PRMT2 catalyzes H3R8me2a (H3R8 asymmetric methylation) on the promoter of BCL2 and then improves the accessibility toward STAT3, which has been shown to promote BCL2 transcription [38]. However, the relationship between PRMT2 and the arginine methylation of STAT1/STAT6 requires further investigation. Likewise, whether arginine methylation affects STAT1/STAT6 phosphorylation also needs further exploration.

## Conclusions

To summarize, our results indicate that PRMT2 regulates macrophage polarization in vitro. Silencing of PRMT2 promotes M1 polarization mediated by activating STAT1 together with attenuating M2 polarization by inhibition of STAT6. Notably, our findings may provide a potential target for controlling macrophage polarization-associated diseases.

## Methods

### BMDMs isolation and culture

Mice on a C57BL/6 background were purchased from Beijing Vital River Laboratory Animal Technology Co., Ltd. BMDMs were isolated from 8-12-week-old mice. Briefly, mice were anesthetized by intraperitoneal injection with avertin (20 µl per gram of body weight). The mice lost consciousness and were unresponsive by using forceps to clamp their toes after about 2 min. Then mice were euthanized by cervical dislocation, and bone marrow of femurs and tibias was taken and incubated in DMEM (BasalMedia) containing 20 ng/ml M-CSF (Peprotech), 10% FBS (fetal bovine serum, Gibco) and 1% penicillin/streptomycin (Gibco) for 7 days at 37 °C with 5% carbon dioxide then matured into a resting state of M0 cells for subsequent stimulation.

### Macrophage polarization

BMDMs were seeded at 1 × 10^6^ cells per well in 6-well plates. Then, cells were treated with LPS (100 ng/ml, sigma, O55: B5) and IFN-γ (20 ng/ml, Peprotech) for 24 h to generate M1 macrophages or exposed to IL-4 (20 ng/mL, Peprotech) for 24 h for the generation of M2 macrophages. M0 was untreated and served as the negative control.

### Reagents and antibodies

DMEM was purchased from BasalMedia. Penicillin/streptomycin and FBS were acquired from Gibco. TRIzol reagent was obtained from Thermofisher. 5x HiScript II Q RT SuperMix and 2x AceQ Universal SYBR qPCR Master Mix were purchased from Vazyme. Recombinant murine M-CSF, IFN-γ, and IL-4 protein were purchased from Peprotech. The antibodies are listed as follows: p-STAT1 (Abclonal), STAT1 (Abclonal), p-STAT3 (Abclonal), STAT3 (Cell Signaling Technology, CST), p-STAT6 (Abcam), STAT6 (Abclonal), p-ERK (CST), ERK (CST), p-JNK (CST), JNK (CST), p-P65 (CST), P65 (CST), p-P38 (CST), P38 (Abclonal), iNOS (CST), Arg1 (CST), PRMT2 (Novus), β-actin (CST), PE anti-mouse F4/80 (Biolegend), FITC anti-mouse CD86 (Biolegend), APC anti-mouse CD206 (Biolegend).

### RNA interference

BMDMs were seeded and adhered into 6-well plates at 1 × 10^6^ cells per well, then transfected with 50 nM *Prmt2* small interfering RNA (*Prmt2* siRNA) duplexes (with the following siRNA sequences: 5’-CGGGUUCUGUUGUGUUACATT-3’) using jetPRIME® (Polyplus) for 48 h, according to the manufacturer’s instructions. BMDMs transfected with an equal amount of universal nontargeting siRNA (sequences: 5’-UUCUCCGAACGUGUCACGUTT-3’) were regarded as negative control (NC).

### Western blot

The total protein of BMDMs was extracted using RIPA (Beyotime) lysis buffer consisting of 1x protease and phosphatase inhibitors cocktail (Beyotime). Protein lysate concentrations were decided using BCA Protein Assay Kit (EpiZyme). 10% sodium dodecyl sulfate-polyacrylamide gel (SDS-PAGE, EpiZyme) electrophoresis was used to separate proteins, which were subsequently transferred onto polyvinylidene fluoride membranes (PVDF, Millipore) according to the standard procedures. After blocking the membranes with 5% milk (EpiZyme) diluted in TBST at room temperature for 1 h, use the primary antibodies to submerge the membranes and incubate them overnight at 4 °C. An HRP (horseradish peroxidase-conjugated) goat anti-rabbit IgG (H + L) (1:5000, Beyotime) and anti-mouse IgG (H + L) (1:2000, CST) were used as secondary antibodies. All signals were detected using Super ECL Detection Reagent (Yeasen) according to the manufacturer’s instructions by the Amersham™ Imager 600.

### Quantitative polymerase chain reaction (qPCR)

The total RNA of BMDMs was extracted using TRIzol reagent (Thermofisher) and performed reverse transcription with 5x HiScript II Q RT SuperMix (Vazyme). Real-time qPCR amplification of reverse transcription products was performed using 2x AceQ Universal SYBR qPCR Master Mix (Vazyme). The primers of each cytokine and gene are displayed in Table 1.

**Table 1:** Primers for qPCR

Gene	Forward Primer (5’-3’)	Reverse Primer (5’-3’)	
*Tnf-α*	CCCTCACACTCAGATCATCTTCT		GCTACGACGTGGGCTACAG
*Il-1β*	GCAACTGTTCCTGAACTCAACT		ATCTTTTGGGGTCCGTCAACT
*Nos2*	GTTCTCAGCCCAACAATACAAGA		GTGGACGGGTCGATGTCAC
*Il10*	GCTCTTACTGACTGGCATGAG		CGCAGCTCTAGGAGCATGTG
*Arg1*	CTCCAAGCCAAAGTCCTTAGAG		AGGAGCTGTCATTAGGGACATC
*Prmt1*	CTTGGCTAATGGGATGAGCCT		GCGTTGGGCTTCTCACTACTT
*Prmt2*	GGAAGACCCCGTGGACTATG		CCGTGGTTTGTCTCAGGATAAGA
*Prmt3*	CAGAGCACCAAAACACACTGG		TCAGGGTCACAATGAGGGAAC
*prmt4*	TGACATCAGTATTGTGGCACAG		CTGAGGAGCCTAAGGGAATCA
*Prmt5*	CTGAATTGCGTCCCCGAAATA		AGGTTCCTGAATGAACTCCCT
*Prmt6*	ATGTCGCTGAGCAAGAAAAGA		CGGAGTAGCACTCGTAGTACAG
*Prmt7*	GCCAGGTCATCCTATGCCG		GCCAATGTCAAGAACCAAGGC
*Prmt8*	ACGTGGTAGCAATCGAAGACA		GCTCCTTCATGGCAACATCC
*Prmt9*	CCTGAAAGAGTGTCCCTAGTTGT		TGCAGAAGTAAATGTTCCCATGC

### Flow cytometry (FCM)

BMDMs were digested by trypsin into single-cell suspensions and then used for subsequent staining. After cells were incubated with Fc receptor blocker (1:500) in the dark under 4 °C for 30 min, samples were subjected to antibodies staining at 4 °C for 30 min, rinsed twice, and resuspended in FACS buffer. Subsequently, these samples were subjected to FCM (Beckman) using different antibodies to analyze samples. PE-labeled anti-mouse F4/80, FITC-labeled anti-mouse CD86, and APC-labeled anti-mouse CD206 were stained to perform the analysis.

### Statistical analysis

Results were shown by mean ± SD. All data were analyzed by a Student’s *t*-test using Prism 9.0. Statistical values where *p* < 0.05 were defined as statistically significant.

### Electronic supplementary material

Below is the link to the electronic supplementary material.

Supplementary Material 1

## Data Availability

The datasets used and/or analysed during the current study are available from the corresponding author on reasonable request.

## Abbreviations

PRMT2: Protein Arginine Methyltransferase 2
BMDMs: Bone Marrow-derived Macrophages
IFN-γ: Interferon-γ
qPCR: Quantitative Polymerase Chain Reaction
FCM: Flow Cytometry
STAT1: Signal Transducer and Activator of Transcription 1
TNF-α: Tumor Necrosis Factor-α
IL-4: Interleukin-4
iNOS: Inducible Nitric Oxide Synthase
CD86: Cluster of Differentiation 86
FIZZ1: Found in Inflammatory Zone 1
Arg1: Arginase 1
TGF-β: Transforming Growth Factorβ
PTMs: Post-translational Modifications
MMA: Mono-methyl-arginine
aDMA: Asymmetric Dimethyl-arginine
sDMA: Symmetric Dimethyl-arginine
NF-κB: Nuclear Factor kappa B
Nos2: Nitric Oxide Synthase 2
MAPK: Mitogen-activated Protein Kinase
ERK: Extracellular-signal-regulated Kinase
JNK: C-Jun N-terminal Kinase
JAK: Janus Kinase
