# Supplementary figures and images for: PRMT2 silencing regulates macrophage polarization through activation of STAT1 or inhibition of STAT6
Source: BMC Immunol. 2024 Jan 3;25:1. doi: 10.1186/s12865-023-00593-w (PMC10765854; doi:10.1186/s12865-023-00593-w)

Fig. 2B

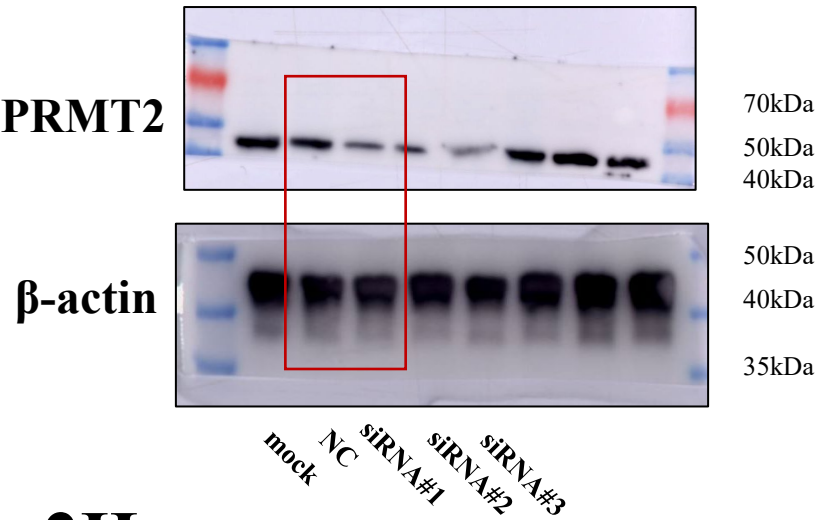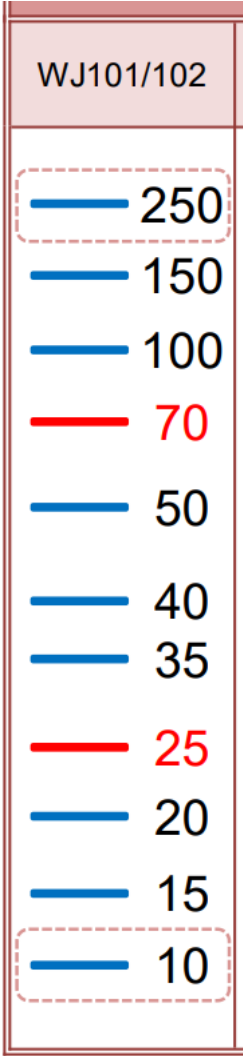

Fig. 2H

siPrmt2 M0 NC M0 siPrmt2 M1 NC M1 siPrmt2 M2 NC M2

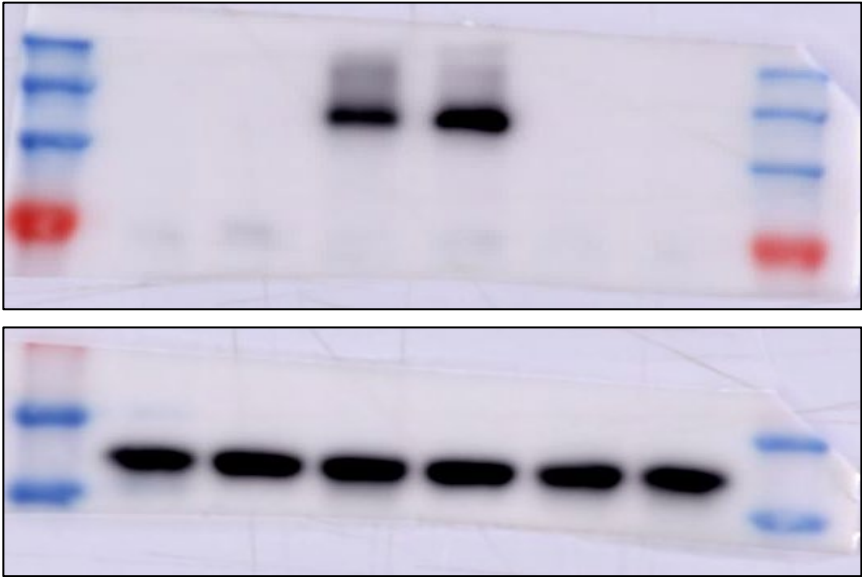

Fig. 3I

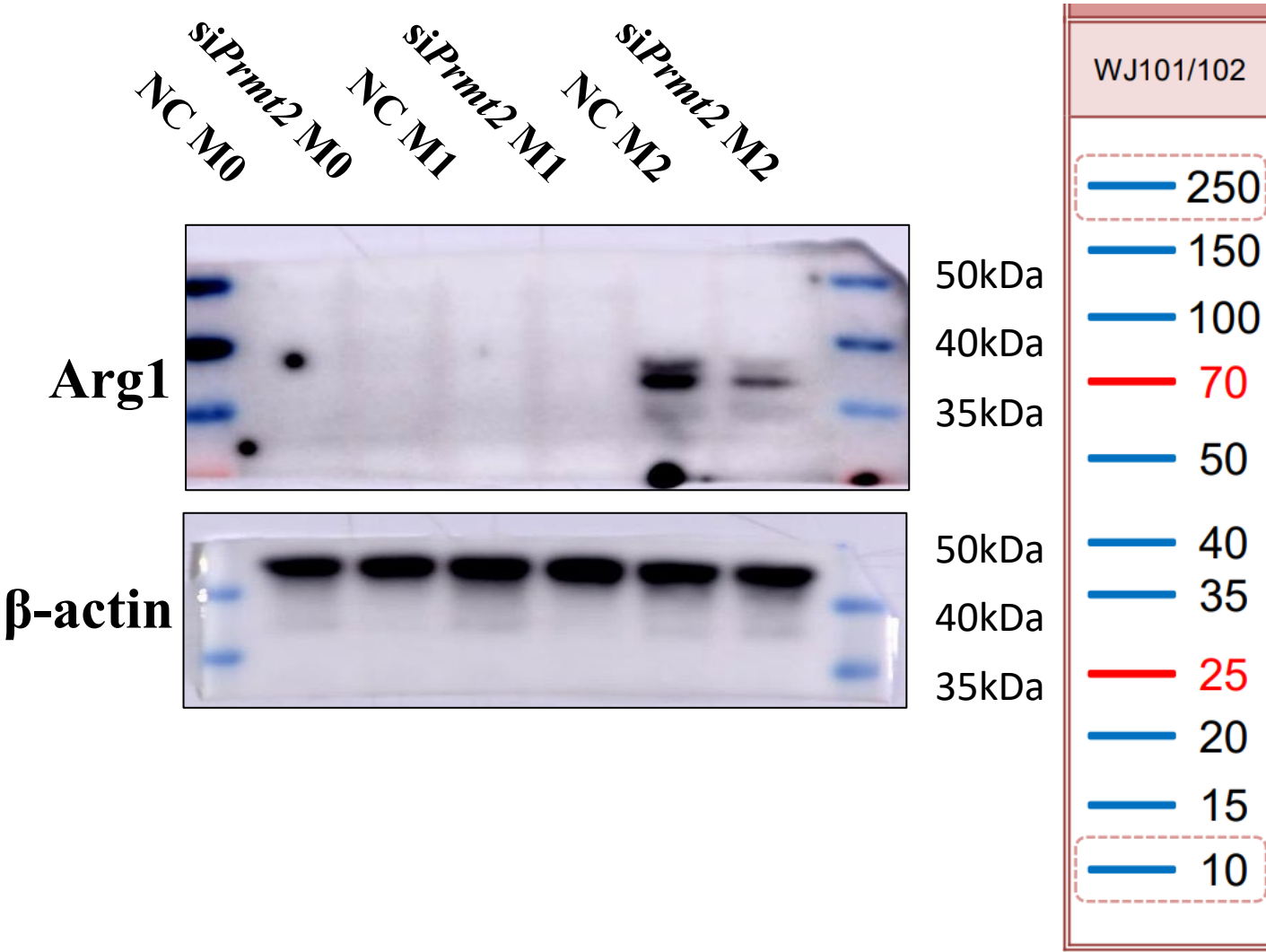

Fig. 4A

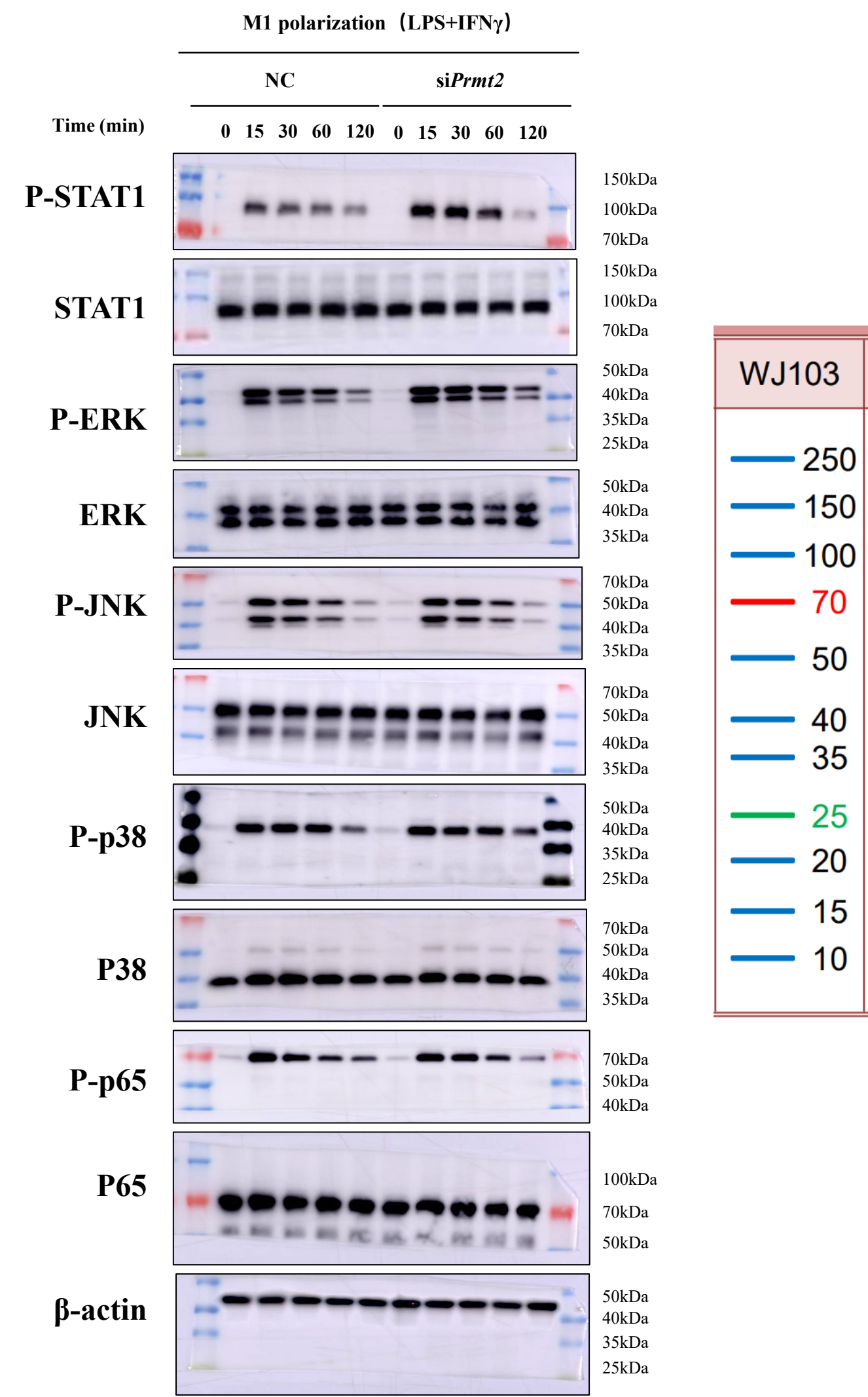

Fig. 5A

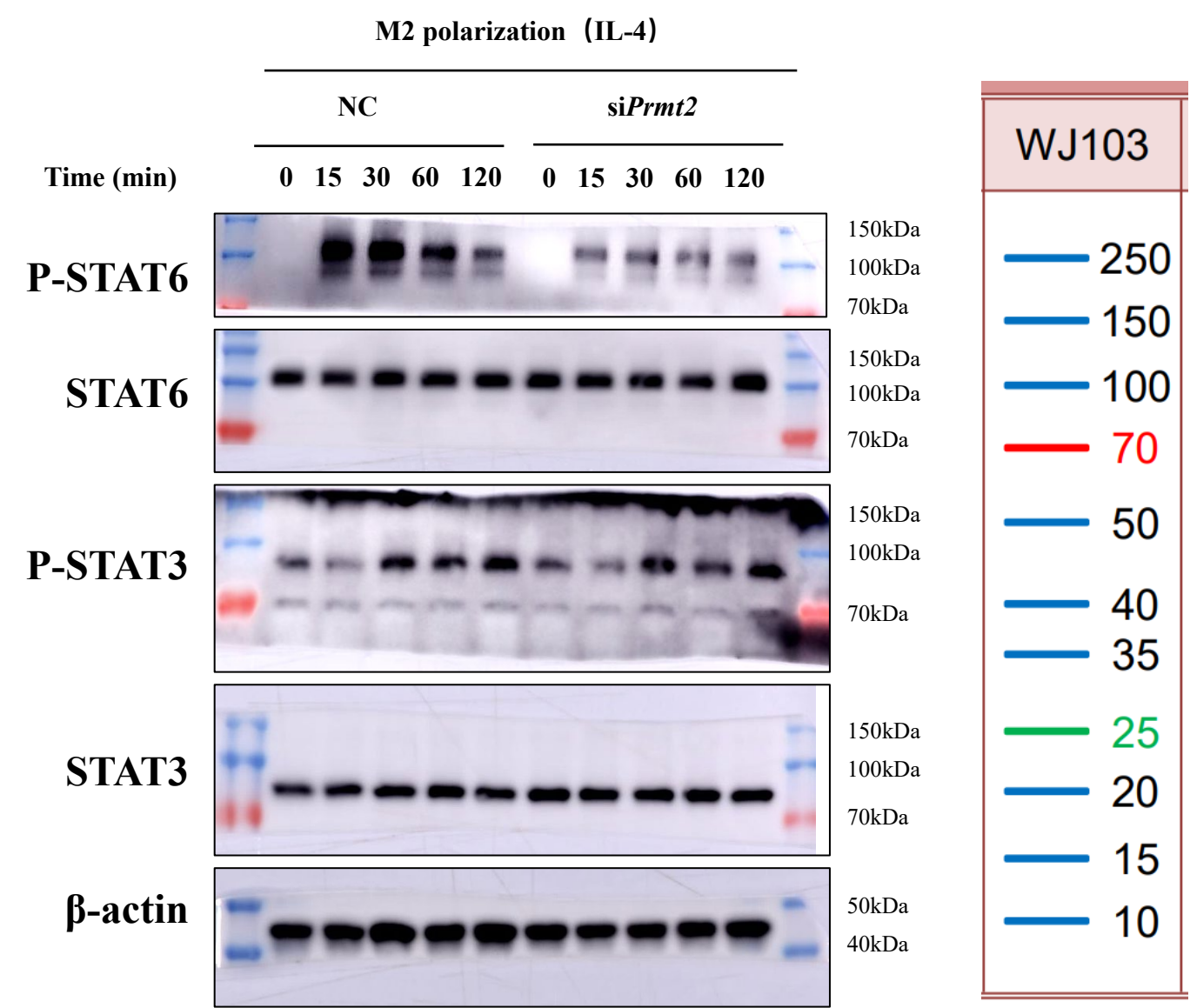

Supplement: Supplementary file 1 — Supplementary Material 1 [file 12865_2023_593_MOESM1_ESM.pdf]
